# Candidate genes for grape white rot resistance based on SMRT and Illumina sequencing

**DOI:** 10.1186/s12870-019-2119-x

**Published:** 2019-11-15

**Authors:** Kai Su, Yinshan Guo, Yuhui Zhao, Hongyan Gao, Zhendong Liu, Kun Li, Li Ma, Xiuwu Guo

**Affiliations:** 1 College of Horticulture, Shenyang Agricultural University, Shenyang, 110866 China; 2 Ministry of Education Key Laboratory of Protected Horticulture, Shenyang, 110866 China

**Keywords:** Grapevine, White rot, SMRT sequencing, Salicylic acid, Jasmonic acid, Resistance gene

## Abstract

**Background:**

White rot is one of the most dangerous fungal diseases and can considerably affect grape berry production and quality. However, few studies have focused on this disease, and thus, finding candidate white rot resistance genes is of great importance for breeding resistant grapevine cultivars. Based on field observations and indoor experiments, the cultivars “Victoria” and “Zhuosexiang” showed significant differences in white rot resistance. For understanding the molecular mechanisms behind it, different phenotypes of grapevine leaves were used for RNA sequencing via Illumina and single-molecule real-time (SMRT) sequencing technology.

**Results:**

A transcript library containing 53,906 reads, including known and novel transcripts, was constructed following the full-length transcriptome sequencing of the two grapevine cultivars. Genes involved in salicylic acid (SA) and jasmonic acid (JA) synthesis pathways showed different expression levels. Furthermore, four key transcription factors (TFs), *NPR1, TGA4, Pti6*, and *MYC2*, all involved in the SA and JA signal pathways were identified, and the expression profile revealed the different regulation of the pathogenesis related protein1 (PR1) resistance gene, as mediated by the four TFs.

**Conclusions:**

Full-length transcript sequencing can substantially improve the accuracy and integrity of gene prediction and gene function research in grapevine. Our results contribute to identify candidate resistance genes and improve our understanding of the genes and regulatory mechanisms involved in grapevine resistance to white rot.

## Background

Grapevine (*Vitis* spp., family Vitaceae) is a perennial woody vine with a history of cultivation extending over 8000 years [1]. Due to its ability to adapt to different environments and high economic and social values, grapevine has been cultivated worldwide. Based on International Wine and Vine Organization data, the cultivation areas of grapevine reached 8.7 million hectares in 2017 [2]. According to the statistical data of the Food and Agriculture Organization of the United Nations (FAO), China ranked first in global grape berries production with 13.1 million tons, and accounted for 15.1% of grape berry worldwide production in 2017 (http://www.fao.org/faostat/zh/#home). In China, *Vitis vinifera* L. is the major table grape berry species, but, due to the high temperature and precipitation that are characteristic of the East Asian monsoon climate, *V. vinifera* cultivars are vulnerable to a variety of fungal diseases [3–5]. Grape white rot (caused by *Coniothyrium diplodiella* (Speg.) Sacc.) is one of the major fungal diseases affecting grapevines. In many grape berry-producing regions affected by this disease, production has been reduced by at least 16.3% [6, 7]. The tissues infected by white rot include leaves, berries, and new shoots. Wounds caused by weather events, insects, and other fungal diseases are the major entry points for the white rot pathogen. In grape berry production, the use of antifungal agents is not recommended, as it causes serious environmental pollution and food safety problems. At present, many grapevine cultivar resources are resistant to white rot disease, comprising powerful resources for resistance genes identification and baseline information for white rot resistance breeding.

In nature, plants can convert light energy into carbohydrates and energy for their development, and these carbohydrates are also the infection targets several microorganisms, including biotrophic, hemibiotrophic, and necrotrophic species [8]. Plants have evolved sophisticated mechanisms of pathogen recognition and defense. Pattern recognition receptor (PRR)-triggered immunity (PTI) is the first tier of plant immunity in systemic acquired resistance (SAR), which is mediated through the recognition of pathogen-associated molecular patterns (PAMPs), and is very effective against most pathogens [9–11]. However, pathogens can synthesize effector proteins and release them into plant cells, counteracting the induction of PTI and enhancing their survival in the host cell. Plants have evolved resistance genes to respond to this effector-triggered susceptibility; these genes recognize effectors and mediate effector-triggered immunity (ETI), the second tier of plant immunity [12–14]. Pathogenesis-related proteins (PRs) are induced by biotic and abiotic stresses and play crucial roles in plant SAR [15]; following infection by pathogenic bacteria, the expression of PRs can enhance plant resistance [16]. Pathogenesis-related 1 (PR1) is a major disease resistance response protein in the PR family involved in plant protection against environmental stresses [14, 17]. Salicylic acid (SA) and jasmonic acid (JA) play crucial roles in plant SAR, and the expression mechanism of PR1 is regulated by a complex mechanism involving several enzymes and transcription factors (TFs) in these pathways [18–21].

Next-generation sequencing (NGS), which is a high-throughput and low-cost technology, has greatly facilitated the development of genomics. However, a major challenge in NGS is the short length of the obtained reads [22–25]. Single-molecule real-time (SMRT) sequencing from Pacific Biosciences (Menlo Park, CA, USA), belongs to the third-generation sequencing [26], provides a possible solution to this shortcoming. With SMRT sequencing, it is possible to achieve full-length reads based on real-time imaging of fluorescently tagged nucleotides as they are synthesized along individual DNA template molecules [27], and it can be widely used for identifying novel genes and transcripts [28, 29]. In the present study, two grapevine cultivars with different resistance to white rot, namely “Victoria” (VT, *V. vinifera*) and “Zhuosexiang” (ZX; *V. vinifera* × *V. labrusca* L.), were infected with white rot spores following an in vitro leaf culture method. Different infection periods (0 h and 72 h) were used for grapevine leaves from the two cultivars, which were then subject to RNA sequencing (RNA-Seq) analysis based on the Illumina (San Diego, CA, USA) X Ten and Pacific Biosciences Sequel platforms. The important white rot resistance gene *PR1* and its regulatory network mediated by SA and JA were identified. Genes involved in these two pathways provide the reference genes required for grapevine white rot resistance breeding, and the full-length transcripts obtained here will greatly improve the accuracy and integrity of grapevine transcripts’ analyses in future research.

## Results

### Evaluation of white rot resistance

**Fig. 1:**
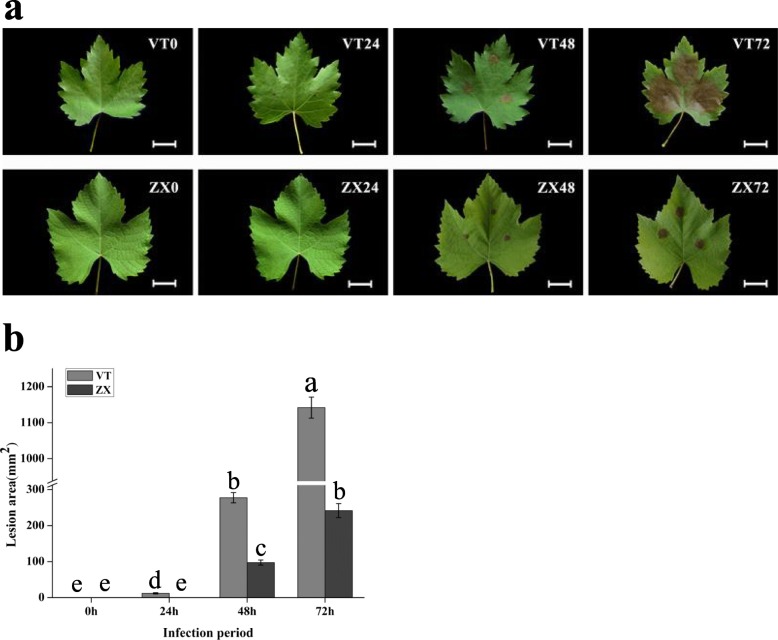
Lesion area identification of VT and ZX after white rot infection. **a** VT and ZX represent grapevine cultivars “Victoria” and “Zhuosexiang”. 0, 24, 48, and 72 represent the hours after infection with white rot. The scale bar is 2 cm. **b** Light-grey bars represent grapevine cultivar VT and dark-grey bars represent grapevine cultivar ZX. The X–axis indicates the infection period of white rot. Error bars represent the standard deviation from three independent experiments. The lowercase letters on the bar chart represent significant differences between two cultivars and different infection period according to Duncan’s multiple range test (DMRT) at *P* < 0.05

Leaves from different branches of cultivars VT and ZX were infected using a white rot spore suspension. At 0, 24, 48, and 72 h post infection, the leaves were evaluated for white rot resistance based on the lesion area. After 72 h, the leaves from the two cultivars presented different degrees of white rot infection (Fig. 1a). The lesion areas of VT leaves were 1141.86 ± 29.13 mm^2^, which was significantly higher than that of ZX (241.88 ± 19.51 mm^2^) (Fig. 1b). Our results showed that white rot had a strong ability to infect grapevine leaves and that ZX showed higher resistance to white rot, compared with VT.

### Illumina and SMRT sequencing data analyses

Grapevine leaves at 0 and 72 h post infection were used for RNA-Seq analysis. Twelve samples (three replicates for each infection period) from each cultivar were used for Illumina sequencing. After removing low quality reads and trimming adapter sequences, 321,687,667 clean reads were obtained (Additional file 1: Table S1). The Pacific Biosciences Sequel platform was used for SMRT sequencing with two SMRT cells. The 647,947 circular consensus sequence (CCS) reads obtained included 569,624 full-length non-chimeric (FLNC) reads and 78,323 no-FLNC reads. The average length of the FLNC reads was 1059 bp. Proovread software was used for the correction of FLNC reads based on Illumina sequencing data [30], and 493,335 FLNC reads were retained and used for further analyses (Additional file 2: Table S2).

### Gene structure and function annotation

**Fig. 2:**
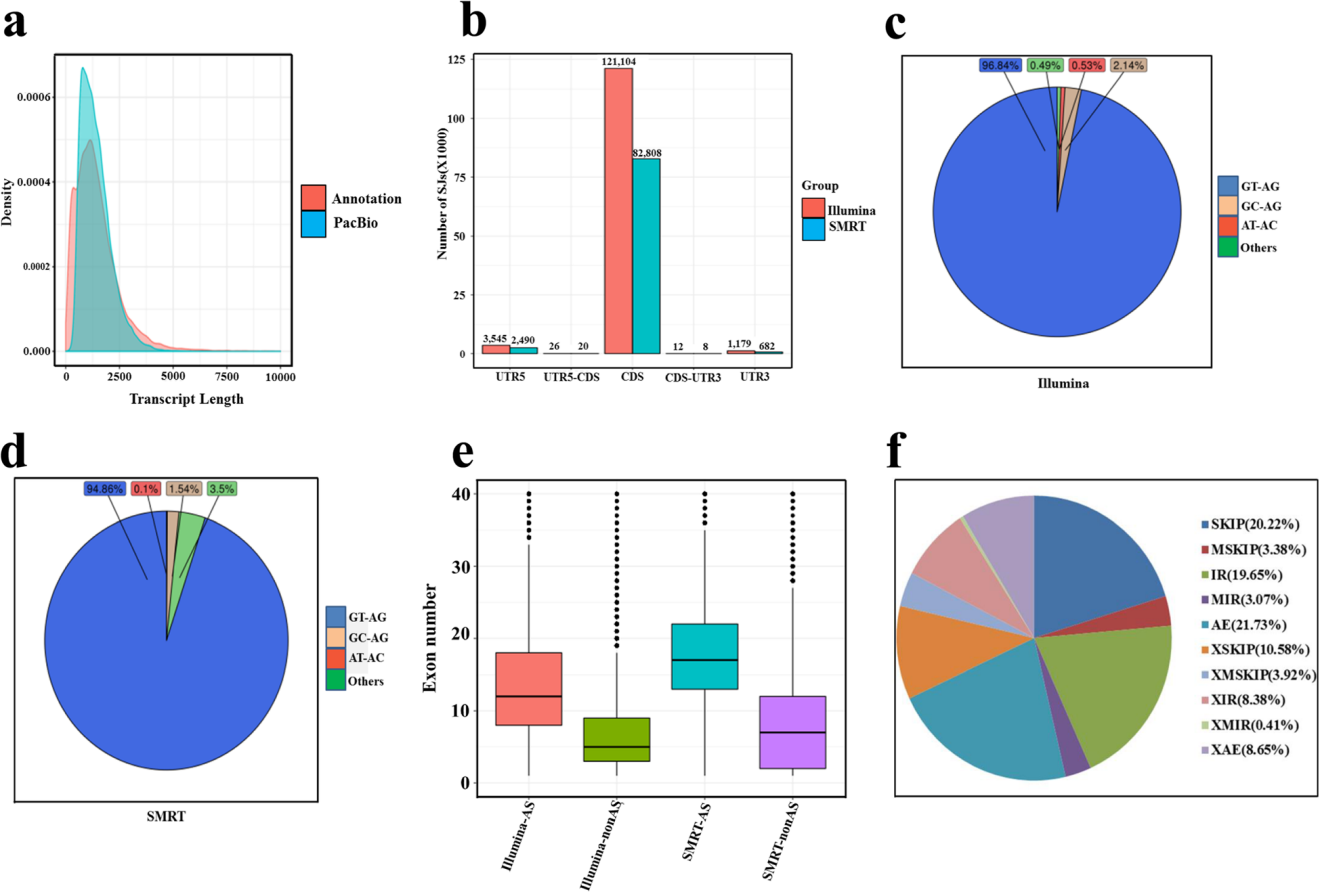
Quality evaluation of SMRT data. **a** Density plot of the length of all previously annotated genes and all SMRT FLNCs. **b** Distribution of splicing junctions along the annotated loci. X–axis represent different gene regions, Y–axis represent the number of Splicing Junction (SJ). **c** and **d** Pie chart showing the percentage of the splicing donor-acceptor di-nucleotide utilization among all transcripts in SMRT and Illumina datasets. **e** Exon numbers of alternate splicing (AS) and non-AS genes in SMRT and Illumina. **f** Percentage of different AS types. Different color represents different AS event

The *V. vinifera* genome based on the Pinot Noir inbred line PN40024 obtained in 2007 contains 26,346 annotated transcripts (http://www.genoscope.cns.fr/ externe/GenomeBrowser/Vitis/). The 493,335 FLNC reads obtained in the present study were used for identifying gene loci and transcripts based on the reference genome. The removal of redundant transcripts reduced transcript number to 37,010, corresponding to 18,698 gene loci, and each transcript represented a unique full-length transcript. Overall, 14,699 gene loci were annotated in the grapevine genome previously, and 3999 gene loci and 27,560 transcripts were first identified in our study based on SMRT sequencing (Table 1). The length and exon number of the new isoforms (Additional file 3: Data S1; the start and end sites of each exon are represented as “,” and the different exons of each isoform as “;”), as well as their annotations and expression levels [in fragments per kilobase of exon model per million reads mapped (FPKM) values] (Additional files 4 and 5: Data S2 and Data S3, respectively), were obtained. The number of transcripts ranging between 0 and 2500 bp, produced via SMRT sequencing, was significantly higher than that of the reference genome (Fig. 2a). For the SMRT sequencing data, the median exon size was 122 bp, which was identical to that of the reference genome. The median gene size and average number of coding exons per gene were 4080 bp and 10.13, respectively, which were higher than those of the reference genome. Alternative splicing (AS) is one of the factors determining the diversity of proteins involved in development and stress responses [31, 32]. In both the SMRT and Illumina datasets, most of the splicing junctions (SJs) resided in the coding sequences (CDS), indicating the potential of AS to affect protein products (Fig. 2b). Most of the splicing donor-acceptor sites were canonical GU-AG sites (96.84% for Illumina and 94.86% for SMRT) (Fig. 2c,d), followed by GC-AG with 2.14% in Illumina and 1.54% in SMRT(Fig. 2c,d). AU-AC splice sites only account for 0.53% in Illumina and 0.1% in SMRT(Fig. 2c,d), as also called U12-type introns, it was reported to have important regulatory role [33]. Interestingly, the exon number of AS genes was higher than that of non-AS genes (Fig. 2e). Among these AS types, exon skipping had the largest gene number (SKIP, 6376), followed by alternative exon ends (AE, 5084) and intron retention (IR, 5272) (Fig. 2f and Additional file 7: Data S4). For the known genes, 3275 were identified underwent alternative splicing, the isoform number and ID of each gene were shown in Additional file 6: Data S5, transcript GSVIVT01031973001 had 34 isoforms and encoded galactinol synthase, and gene GSVIVT01031973001 had 30 isoforms and encoded the glycine-rich RNA binding protein GRP2A. We also found 1841 long non-coding RNAs (LncRNAs) (Additional file 8: Data S6), accounts for 6.47% of all novel isoforms; 76 fusion genes (Additional file 9: Data S7), the number of different fusion types (Inter-chromosome and Intra-chromosome) were 38 respectively. Besides that, we also identified 22,638 polyA sites from 9039 genes, 5364 of which presenting alternative polyadenylation (APA) (Additional file 10: Data S8).

**Table 1:** Statistics of gene loci and isoforms for SMRT sequencing data

Categories	Number of Loci	Number of isoforms
Known genes and isoforms	2381	9450
Known genes and new isoforms	12,318	23,001
New genes and isoforms	3999	4559
Total genes and isoforms	18,698	37,010

We constructed a new transcript library containing 53,906 transcripts (26,346 from reference genome and 27,560 novel isoforms from novel and known loci) after the combination of novel transcripts identified by SMRT sequencing and annotated transcripts from the reference genome (Additional file 11: Data S9). All these transcripts were then used to search against the National Center for Biotechnology Information (NCBI) non-redundant (NR), Swiss-Prot, gene ontology (GO), and clusters of euKaryotic orthologous genes (KOG) protein databases and Kyoto Encyclopedia of Genes and Genomes (KEGG) pathway database, the details of which are shown in Table 2.

**Table 2:** Summary of transcripts annotated in different database

Database	Number	Percentage (%)
NR	51,866	96.22
Swiss-Prot	40,363	74.88
GO	35,965	66.72
KEGG	22,148	41.09
KOG	17,753	32.93
Total	53,906	100

### Gene expression analysis based on Illumina and SMRT data

**Fig. 3:**
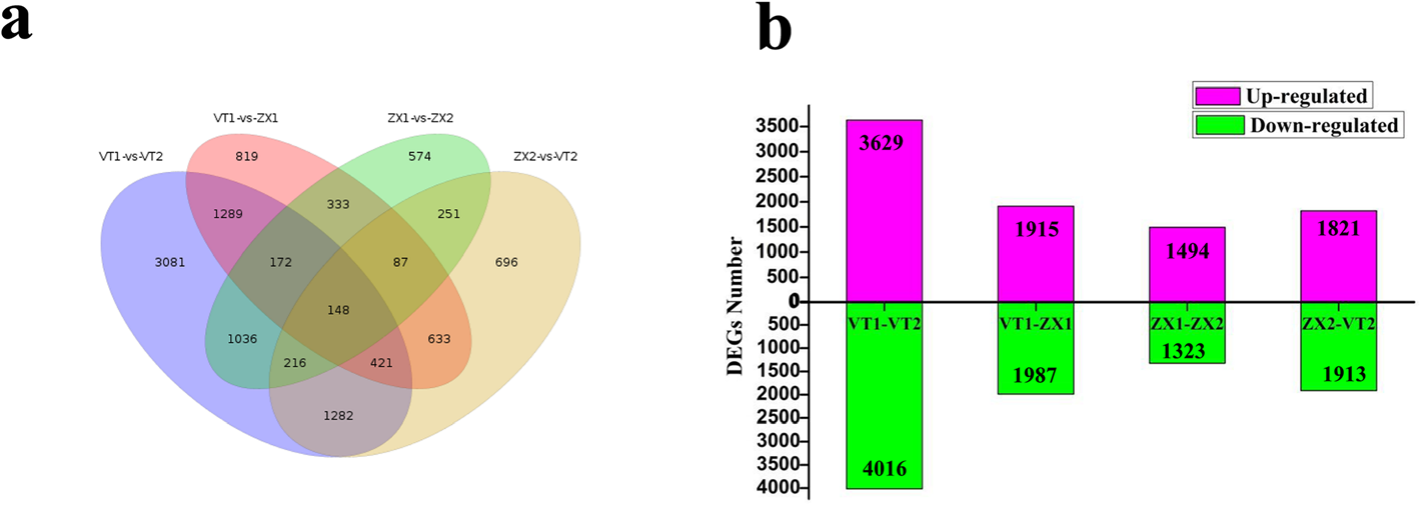
Statistics and Venn diagram analysis of DEGs in different cDNA libraries. Pink and green colors represent up-regulated and down-regulated DEGs, respectively. VT1 and VT2 represent DEGs of VT at 72 h relative to DEGs at 0 h; ZX1 and VT1 represent DEGs of VT at 0 h relative to DEGs of ZX at 0 h; ZX1 and ZX2 represent DEGs of ZX at 72 h relative to DEGs of ZX at 0 h; ZX2 and VT2 represent DEGs of VT at 72 h relative to DEGs of ZX at 72 h

**Fig. 4:**
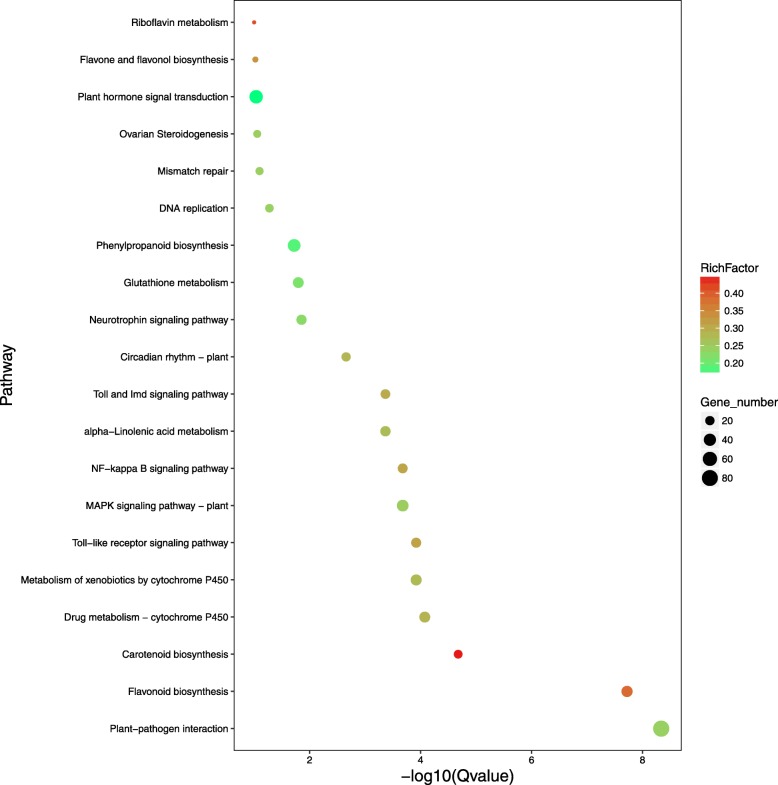
Enrichment of KEGG pathways in VT vs. ZX at 72 h post infection. The colored area represents the number of genes involved in each pathway, and the color intensity represents the enrichment factor. The Q value represents the multiple hypothesis testing of *P*-values

The 321,687,667 clean reads produced by Illumina sequencing were aligned to the newly constructed transcript library using Bowtie software [34], and the matching rate is shown in Additional file 12: Table S3. The gene expression patterns of VT1, VT2, ZX1, and ZX2 samples were calculated using the FPKM values and the RSEM software [35]. The differentially expressed genes (DEGs) were statistically evaluated using the DESeq method [36]. Our results revealed that 7645 DEGs were discovered in VT1 vs. VT2, 2817 in ZX1 vs. ZX2, 3902 in VT1 vs. ZX1, and 3734 in VT2 vs. ZX2; 148 DEGs were common to the four libraries (Fig. 3a,b). The VT1 vs. VT2 and ZX1 vs. ZX2 comparisons revealed more down-regulated than up-regulated genes in VT and more up-regulated than down-regulated genes in ZX after white rot infection. The white rot-susceptible VT cultivar showed more DEGs during infection than the white-rot resistant cultivar ZX, indicating that grapevine white rot is more successful at modifying leaf metabolism in the susceptible form. Based on VT1 vs. ZX1 and ZX2 vs. VT2 comparisons, there were more genes down-regulated genes in ZX than in VT at 0 h post infection, and more up-regulated genes in ZX than in VT at 72 h post infection, indicating that more white rot resistance genes were highly expressed in the resistant cultivar after infection. Using the KEGG pathway database to search the functional networks of biological interactions, we assigned 2078 DEGs to 263 KEGG pathways. The top 20 enriched KEGG pathways are shown in Fig. 4 and Additional file 13: Data S10.

### Differential expression analyses of candidate genes

**Fig. 5:**
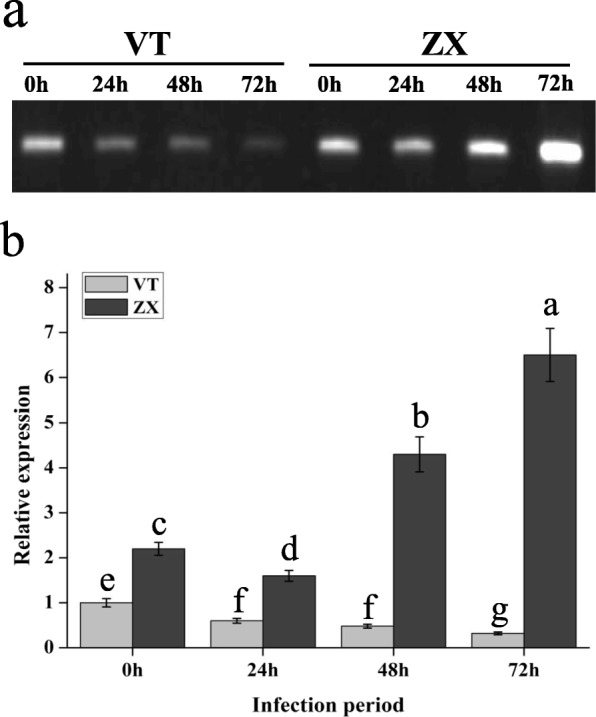
Semi-quantitative PCR and qRT-PCR analyses of *PR1* expression at different infection periods. Light-grey bars represent cultivar VT and dark-grey bars represent cultivar ZX. Error bars represent the standard deviation of three biological replicates. The lowercase letters on the bar chart represent significant differences between two cultivars and different infection period according to Duncan’s multiple range test (DMRT) at *P* < 0.05

**Fig. 6:**
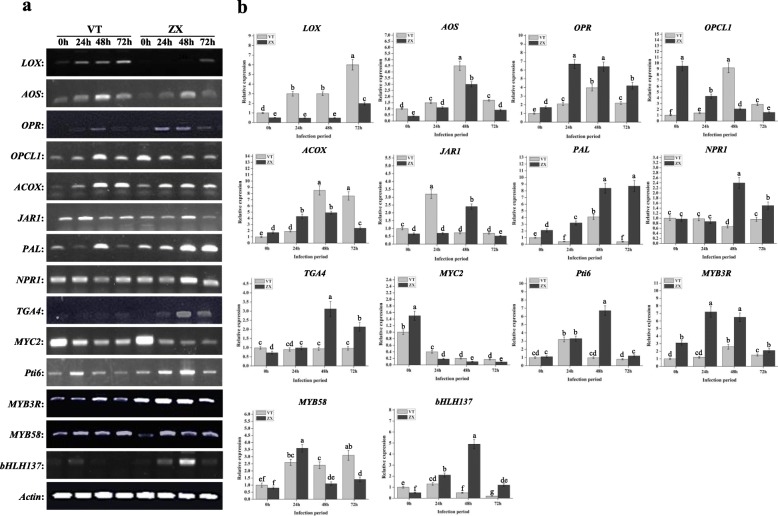
Semi-quantitative PCR and qRT-PCR analysis of genes involved in JA and SA synthesis and signal pathways. Light-grey bars represent cultivar VT and dark-grey bars represent cultivar ZX. The X–axis indicates the infection period of white rot. Error bars represent the standard deviation of three biological replicates. The lowercase letters on the bar chart represent significant differences between two cultivars and different infection period according to Duncan’s multiple range test (DMRT) at *P* < 0.05. Abbreviations: *LOX*: lipoxygenase; *AOS*: allene oxide synthase; *OPCL1*: OPC-8:0 CoA ligase 1; *ACOX*: acyl-CoA oxidase; *OPR*: 12-oxophytodienoic acid reductase; *PAL*: phenylalanine ammonia-lyase; *JAR1*: jasmonic acid resistance 1; *NPR1*: non-expressor of pathogenesis related gene 1. *TGA4*: bZIP transcription factor 4; *MYC2*: bHLH zip-type transcription factor; *Pti6*: ERF transcription factor; *MYB3R* and *MYB58*: MYB transcription factors; *bHLH137*: bHLH transcription factors

**Fig. 7:**
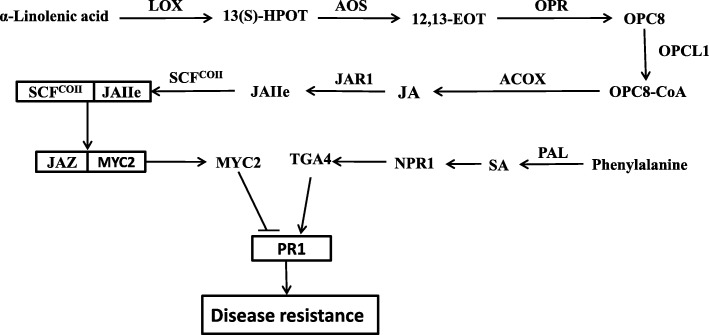
Regulatory network of *PR1* gene expression mediated by JA and SA signaling pathways. Abbreviations: *LOX*: lipoxygenase; *AOS*: allene oxide synthase; *OPCL1*: OPC-8:0 CoA ligase 1; *ACOX*: acyl-CoA oxidase; *OPR*: 12-oxophytodienoic acid reductase; *PAL*: phenylalanine ammonia-lyase; *JAR1*: jasmonic acid resistance 1; *NPR1*: non-expresser of pathogenesis related gene 1. *TGA4*: bZIP transcriptional regulator 4; *MYC2*: bHLH zip-type translation factor

The KEGG enrichment analysis evidenced many differently enriched genes in the “MAPK signaling” (*P*-value ≤6.34E^− 6^), “plant-pathogen interaction” (*P*-value ≤1.73E^− 11^), and “alpha-linolenic acid metabolism” (P-value ≤1.51E^− 5^) pathways at 72 h post infection (Fig. 4). According to our results, the grape *PR1* gene (GSVIVT01038540001, Additional file 11: Data S9) was differentially expressed in both the “MAPK signaling” and “plant-pathogen interaction” pathways. After quantitative real-time (qRT) and semi-quantitative PCR analyses, the expression of *PR1* was down-regulated in VT after white rot infection and there is a decreased expression at 24 h post infection in ZX and then up-regulated from 48 h to 72 h (Fig. 5). In plant SAR, the regulation mechanism of *PR1* involves a complex network, and *PR1* expression is mainly induced by SA and JA. The expression of genes related to JA and SA synthesis and signal pathway were identified by the qRT-PCR and semi-quantitative PCR analyses. The result showed that, after white rot infection, several key enzyme genes located upstream JA synthesis were up-regulated in both VT and ZX. The expression level of *lysyl oxidase* (*LOX*; GSVIVT01025340001, Additional file 11: Data S9) continuously increased in both ZX and VT, peaking at 72 h post infection; in addition, the expression level in VT was higher than that in ZX. *Allene oxide synthase* (*AOS*; GSVIVT01009616001, Additional file 11: Data S9) and *acyl-coenzyme A oxidase* (*ACOX*; GSVIVT01016325001, Additional file 11: Data S9) were also induced after infection and their expression levels peaked at 48 h post infection; the expression level of these two genes in VT was higher than that in ZX from 48 to 72 h post infection. The expression of *12-oxo-phytodienoic acid reductase* (*OPR*; GSVIVT01013386001, Additional file 11: Data S9) was induced in both ZX and VT, and its expression peaked from 24 to 48 h post infection in ZX and at 48 h post infection in VT, and it was higher in ZX than in VT. Gene *OPC-8:0 CoA ligase 1* (*OPCL1*; GSVIVT01008694001, Additional file 11: Data S9) was repressed in ZX at the onset of white rot infection, but induced in VT peaking at 48 h post infection. As a key enzyme-coding gene involved in SA synthesis, *PAL* (GSVIVT01006148001, Additional file 11: Data S9) was up-regulated and peaked at 48 h post infection in both ZX and VT, and its expression level was higher in ZX than in VT. The expression of genes and TFs located downstream the JA and SA pathways were also identified. In the JA pathway, *jasmonic acid resistance 1* (*JAR1*; GSVIVT01030558001, Additional file 11: Data S9) was up-regulated in both ZX and VT and peaked at 24 h post infection for VT and 48 h post infection for ZX. Transcription factor *MYC2* (GSVIVT01027162001, Additional file 11: Data S9) was repressed after white rot infection, although its expression level was higher in VT than in ZX from 24 to 72 h post infection. Regarding the SA pathway, *NPR1* (GSVIVT01015181001, Additional file 11: Data S9) and *TGA4* (GSVIVT01033632001, Additional file 11: Data S9) were significantly up-regulated in ZX and peaked at 48 h post infection; in VT, *NPR1* was repressed at 48 h after infection, and no significant difference was evidenced for *TGA4*. The expression level of these two genes was higher in ZX than in VT from 48 to 72 h post infection (Fig. 6). These results indicated that SA and JA may mediate the expression of *PR1* in opposite directions after white rot infection (Fig. 7).

Some TFs, which may play important roles in plant development and resistance, presented large fold-changes in their expressions in VT2 vs. ZX2 comparisons. Two *MYB* TFs and one *basic helix-loop-helix* (*bHLH*) TF were differentially expressed after white rot infection. Whereas *MYB3R* (GSVIVT01027493001, Additional file 11: Data S9) and *bHLH137* (GSVIVT01008628001, Additional file 11: Data S9) were both up-regulated in ZX and VT with higher expression levels in ZX than in VT from 24 to 72 h post infection, *MYB58* (GSVIVT01036802001, Additional file 11: Data S9) was induced in ZX and VT after white rot infection, but its expression level peaked at 24 h post infection for ZX and at 72 h post infection for VT (Fig. 6).

## Discussion

Plant hormones are small signal molecules; in addition to regulate plant development, they also play an important role in plant defense against biotic and abiotic stresses via SAR. Many studies have highlighted the role of *PR1* in SAR for resistance against pepper Phytophthora disease and bacterial wilt [37], powdery mildew in barley [38], and Phytophthora disease and gray mold in tobacco [18, 39], among others. In the present study, grapevine white rot, caused by a canonical necrotrophic pathogen, induced a series of genes and TFs including *LOX*, *AOS*, *OPR*, *OPCL1*, *ACOX*, *JAR1*, *PAL, NPR1, TGA4*, and *MYC2* in JA and SA synthesis and signal pathways.

An increasing number of studies have focused on the crosstalk between SA and JA signals in plant SAR, including the mediation of *PR1* expression [17, 40–42]. Jasmonate ZIM-domain (JAZ) proteins play important roles in the JA signal pathway. Without environmental stress, JAZ proteins can bind *MYC2* and inhibit its regulatory function. In the SA signal pathway, *NPR1* and *TGA* interaction is required for positively promoting the activity of *PR* mediated by SA [20, 21]. According to the present results, the interaction between *NPR1* and *TGA4* may play an important role in *PR1* expression and white rot resistance. Gu et al. indicated that SA could induce the expression of *PR1* through *Pti4/5/6* TFs and enhance plant defense to *Erysiphe orontii* and *Pseudomonas syringae* pathovar *tomato* [18]. Interestingly, in *Arabidopsis thaliana,* the ethylene response factor (ERF) *AtEBP* can interact with *TGA* and then regulate the expression of *PR* genes [43]. Here, we found that *Pti6* (chr6.849.1, Additional file 11: Data S9), a TF of the ERF family induced after white rot infection and higher expressed in ZX than in VT (Fig. 6). Based on our results, the interaction between *TGA4* and *Pti6* may also play a crucial role in promoting *PR1* expression in white rot resistance.

According to the results obtained here, *MYC2, NPR1*, *TGA4*, and *Pti6* may play crucial roles in *PR1* expression, while SA and JA signal pathways showed antagonistic roles in the regulation of these TFs. Spoel et al. found that, as a key regulatory factor in the SA signal pathway, *NPR1* could suppress the expression of *LOX,* which is involved in JA synthesis, thereby repressing the effect of JA on *PR1* expression [44]. However, Li et al. indicated that the interaction between *MdMYC2* and *ERF2* suppresses the regulatory effect of *ERF2* on its target gene in ethylene synthesis [45]. Thus, the interaction between *MYC2* and *Pti6* may also play important role in *PR1* expression and white rot resistance. Overall, transcription factor *MYC2* negatively regulates grapevine white rot resistance whereas TFs *NPR1*, *TGA4*, and *Pti6* positively regulate it. The results obtained so far indicate that the regulatory mechanisms of these TFs on *PR1* expression are complex and important in grapevine white rot resistance.

Transcription factors within the *MYB* superfamily have a conserved *MYB* domain and play an important role in mediating plant development and response to environmental stresses. In *A. thaliana*, *AtMYB96* mediates the defense against bacterial infection by inducing SA biosynthesis [46], *AtMYB30* is involved in the resistance and associated cell death responses to bacterial infections through the transcriptional activation of very-long-chain fatty acid metabolism [47], and *AtMYB44* plays a critical role in resistance against the phloem-feeding generalist green peach aphid (*Myzus persicae* Sulzer) and leaf-chewing specialist diamondback moth (*Plutella xylostella* L.) larvae [48]. Many other *MYB* TFs, such as *AtMYB13, AtMYB15, AtMYB33, AtMYB70, AtMYB73, AtMYB77*, and *AtMYB101*, have been reported as involved in *A. thaliana* defense against environmental stresses [49, 50]. To date, there have been no reports on the *MYB* TFs mediating grapevine defense to white rot. Here, two *MYB* TFs (*MYB3R* and *MYB58*) were induced by white rot infection in grapevine leaves, especially *MYB3R*, which showed a higher expression level in ZX than in VT.

Transcription factors within the *bHLH* superfamily also mediate plant resistance. Under abiotic stress, *bHLH122* can improve *A. thaliana* resistance to drought and osmotic stress [51]. Several studies have shown that, under biotic stress, *bHLH25, bHLH27*, and *bHLH060* are negative regulators of *A. thaliana* defense against the cyst nematode *Heterodera schachtii* Schmidt. and *Pseudomonas syringae* [52, 53]. In the present study, *bHLH137* was significantly induced in VT and ZX after white rot infection, thus revealing that this TF might be a positive regulator in grapevine white rot defense.

The KEGG pathway analysis performed here revealed many DEGs were enriched in the top-six categories, in terms of the -log10 Q value. The “carotenoid biosynthesis” pathway showed the highest enrichment, and DEGs enriched in this pathway were related to *15-cis-phytoene synthase* (crtB), *beta-carotene isomerase* (DWARF27), *beta-carotene 3-hydroxylase* (crtZ), *zeaxanthin epoxidase* (ZEP), *capsorubin synthase* (CCS1), *9-cis-epoxycarotenoid dioxygenase* (NCED), *abscisic-aldehyde oxidase* (AAO3), *(+)-abscisic acid 8′-hydroxylase* (CYP707A) and *abscisate beta-glucosyltransferase* (AOG) synthesis. The DEGs enriched in the “drug metabolism - cytochrome P450” and “metabolism of xenobiotics by cytochrome P450” pathways were related to *dimethylaniline monooxygenase* (FMO), *glutathione S-transferase* (GST), *cytochrome P450 family 1 subfamily A polypeptide 1* (CYP1A1). In the “toll-like receptor signaling pathway”, enriched DEGs were related to *lipopolysaccharide-binding protein* (LBP), *interleukin-1 receptor-associated kinase 1* (IRAK1), and *interleukin-1 receptor-associated kinase 4* (IRAK4). In the “flavonoid biosynthesis” pathway, enriched DEGs were related to *chalcone synthase* (CHS), *flavonoid 3′-monooxygenase* (CYP75B1), *bifunctional dihydroflavonol 4-reductase/flavanone 4-reductase* (DFR), *naringenin 3-dioxygenase* (F3H), *flavonoid 3′,5′-hydroxylase* (CYP75A), *anthocyanidin synthase* (ANS), *leucoanthocyanidin reductase* (LAR), and *anthocyanidin reductase* (ANR). The functions of most of the abovementioned DEGs have been studied, although focusing mainly on plant secondary metabolic reactions, as is the case of the DEGs in the carotenoid and flavonoid biosynthesis pathways, and CYP450-related DEGs [54–68]. The DEGs enriched in the “plant-pathogen interaction” pathway were mostly involved in hypersensitive response, such as the DEGs related to *respiratory burst oxidase* (RBOH) [69, 70] and *heat shock protein 90* (HSP90) [71], and their expression level were higher in ZX than in VT after white rot infection. Overall, the expressions of most DEGs enriched in the abovementioned pathways were higher in ZX than in VT, especially that of the DEGs in the “carotenoid biosynthesis” and “flavonoid biosynthesis” pathways. This suggested that, in addition to the genes involved in plant SAR, some genes involved in plant secondary metabolite biosynthesis might play important roles in grapevine white rot resistance. However, this regulatory network is complex and needs further research.

## Conclusions

We conducted SMRT and Illumina sequencing of the grapevine transcriptome following a biotic stress (white rot infection). The SMRT data revealed 18,698 gene loci and 37,010 full-length transcripts, including 3999 novel gene loci and 30,860 novel transcripts. By combining these new transcripts with that annotated in the grapevine reference genome, we constructed a new library containing 53,906 transcripts. The new full-length transcripts obtained here provide important reference full-length transcript resources that help identifying new resistance genes in grapevine, especially those involved in biotic stresses response. We also found that *PR1* expression, mediated by the crosstalk of SA and JA, is crucial in grapevine white rot resistance. The candidate genes revealed in the present study enrich our understanding of and provide basis for grapevine white rot resistance breeding.

## Methods

### Plant materials and white rot infection

Young and healthy leaves of the “Victoria” (*V. vinifera*) and “Zhuosexiang” (*V. vinifera* × *V. labrusca* L.) cultivars were collected from the grapevine experimental garden of Shenyang Agricultural University, Liaoning Province, P. R. China (E123°24′, N41°50′). *Coniothyrium diplodiella* strain JZB3700001 was obtained from the Beijing Academy of Agriculture and Forestry Sciences, Beijing Key Laboratory of Environmentally Friendly Management of Fruit Diseases and Pests in North China. Surface sterilization of the collected leaves was performed as previously described [72]. After sterilization, the leaves of each cultivar were placed in plastic Petri dishes and punctured on the left, middle, and right regions. A 10-μL 10^6^/mL white rot spore suspension was then dropped on the wounding points to induce white rot infection. All leaves were incubated in a moist chamber at 28 °C with 95% relative humidity. Seventy-two hours later, the infected leaves were used for a white rot infection survey, and the lesion areas of the infected regions were measured using the YMJ-C smart leaf area meter (Tuopu Instrument Co. Ltd., China). Leaves from two infection stages, 0 and 72 h, were then rapidly frozen in liquid nitrogen and stored at − 80 °C for further analyses.

### RNA extraction and sequencing

Total RNAs were extracted from the leaves of the different grapevine cultivars using the Plant Total RNA Isolation Kit (Sangon Biotech, Shanghai, China; No. SK8631), according to the manufacturer’s instructions. The RNA purity and integrity were measured in the NanoDrop 2000 (Thermo Fisher Scientific, Waltham, Ma, USA) and Agilent 2100 (Agilent Technologies, Santa Clara, CA, USA) equipments, respectively.

For SMRT sequencing, pure RNAs of each leaf sample were pooled, and cDNA synthesis was performed using the SMARTer™ PCR cDNA Synthesis Kit (Takara Bio Inc., Mountain View, CA, USA). The reverse transcriptase (RT) begins synthesis at the poly(A) tail of the fragment and then synthesizes a cDNA complementary to the RNA. Full-length cDNA fragments assayed by the BluePippin System were then amplified for a second time. Once the double-stranded cDNA was prepared, the remaining overhangs were converted into blunt ends via exonuclease, and then, a SMRT adaptor with a hairpin loop structure was ligated to the end of the cDNA. The SMRTbell templates were then sequenced on the Pacific Biosciences Sequel System using two SMRT cells. Using SMRT Link v5.0 to conduct raw data preprocessing and filtering, the major parameter were: Minimum Number of Passes = 1; Minimum Predicted Accuracy = 0.8; Minimal Read Score = 0.65.

For Illumina sequencing, the cDNA libraries were constructed for infected grape leaves at 0 and 72 h post infection, using three replicates per cultivar. The cDNA libraries were sequenced on the Illumina X Ten high-throughput sequencing platform, with pair-end reads of 150 bp.

### Identification of gene loci and isoforms

Error correction of FLNC reads with the high quality Illumina short reads was performed using Proovread version 2.12 with the default parameters [30]. After correction, FLNC reads were aligned to the grapevine reference genome (http://www.genoscope.cns.fr/externe/GenomeBrowser/Vitis/) using GAMP software [73]. Isoforms supported with at least two FLNC reads, or one FLNC read with percentage-of-identity (PID) higher than 99%, or all junction sites that were fully supported by Illumina reads or annotations of the grape genome were retained. Isoforms with overlap > 20% (at least one exon overlap > 20%) were considered to be from the same gene locus. New loci and isoforms were identified as follows: 1) no overlap or the overlap region was less than 20% when blast with the genome annotation; 2) the overlap region was more than 20% but the isoform direction was opposite. Known gene novel isoforms were determined as follows: 1) when compared with the genome annotation, one or more new splice sites were discovered in the isoforms; 2) isoform in our study and the annotated transcript of reference genome are not signal exons gene at the same time.

### Alternative splicing events

AS events were classified and characterized by comparing different isoforms of the same gene locus using Asprofile software [74] based on the full length isoforms achieved by SMRT sequencing, the AS events in this study were exon skipping and cassette exons (SKIP, MSKIP), retention of single (IR) and multiple (MIR) introns, alternative exon ends (5′, 3′, or both) (AE), approximate exon skipping (XSKIP) and cassette exons (XMSKIP), approximate retention of single (XIR) and multiple (XMIR) introns and approximate alternative exon ends (XAE).

### Fusion genes, Lnc RNA and alternative polyadenylation (APA) prediction

Fusion genes were identified as follows: 1) the FLNC overlapped two or more gene loci of the reference genome and the overlap region of each gene locus was less than 10 bp; 2) the distance of each gene loci was more than 50 kb in reference genome; 3) FLNC meet with global PID ≥ 10% and local PID ≥90%; 4) each of the gene locus must be supported with at least two Illumina reads. Lnc RNA and APA prediction in this study were according to the introduction [75, 76] by using CPATv1.2.2 and Tapis software.

### Gene function annotation

To understand gene functions, the data produced by SMRT sequencing were annotated using Diamond software against the NCBI NR, Swiss-Prot, and KOG databases [77]. The alignments against the NR database were used in Blast2GO (https://www.blast2go.com/) to obtain GO annotations. The KEGG pathway assignments were performed using KOBAS software [78].

### Differential gene expression analysis and qRT-PCR validation

Illumina data were queried to the newly constructed library (including known and novel isoforms) using Bowtie software [34]. Gene expression levels were calculated via FPKM using RSEM [35]. The DESeq package was used to identify DEGs with a threshold false discovery rate (FDR) < 0.05, and |log_2_ fold-change (FC)| > |1| [35]. Gene ontology and KEGG pathways enrichment analysis were performed using a hypergeometric test and Benjamini-Hochberg multiple testing adjustment. The RNA extraction from each cultivar at each infection stage was performed as described above. The cDNAs were synthesized from RNAs using the PrimeScript™RT Kit (TaKaRa Bio Inc., Kusatsu, Japan; Cat. RR047A). A five-fold dilution of cDNA was used as the template. The reaction solution contained SYBR® PremixExTaq™ II (Tli RNaseH Plus) (TaKaRa; Cat. RR820A) and the qRT-PCR was conducted in an ABI QuantStudio 6 Flex System (Applied Biosystems, Foster City, CA, USA). The relative expression levels of the selected genes, normalized to grapevine *β-actin* [79], were calculated using the 2^-ΔΔCt^ method. All reactions were performed using three biological replicates. The primers for the validation of DEGs are listed in Additional file 14: Table S4.

## Supplementary information

**Additional file 1: Table S1.** Illumina sequencing evaluation and mapping rate.

**Additional file 2: Table S2.** Statistics analysis of SMRT data.

**Additional file 3: Data S1.** Statistics of new isoform length and exon number.

**Additional file 4: Data S2.** Statistics of isoform annotation message.

**Additional file 5: Data S3.** Expression level of isoforms represented by FPKM

**Additional file 6: Data S4.** Different alternative splicing events in grape leaves predicted by ASprofile software.

**Additional file 7: Data S5.** Statistics of isoform number for gene underwent alternative splicing.

**Additional file 8: Data S6.** LncRNAs predicated by CPAT software based on the novel genes and isoforms from SMRT data

**Additional file 9: Data S7.** Fusion genes predicated based on SMRT data

**Additional file 10: Data S8.** Prediction of alternative polyadenylation sites and related transcripts based on SMRT data

**Additional file 11: Data S9.** New transcript library from merging transcripts of known gene model and novel isoforms of SMRT

**Additional file 12: Table S3.** Reads from Illumina sequencing alignment to the new constructed library

**Additional file 13: Data S10.** KEGG enrichment of DEGs between VT2 and ZX2

**Additional file 14: Table S4.** Primers used for semi-quantitative PCR and qRT-PCR validation

## Data Availability

The data supporting the results presented in this article are included as additional files. The raw reads of the two SMRT libraries and 12 Illumina RNA-seq libraries generated in this study have been uploaded to the Sequence Read Archives Database (http://www.ncbi.nlm.nih.gov/sra/) under the accession number SRP151613.

## Abbreviations

AAO3: Abscisic-aldehyde oxidase
ACOX: Acyl-CoA oxidase
AE: Alternative exon ends
ANR: Anthocyanidin reductase
ANS: Anthocyanidin synthase
AOG: Abscisate beta-glucosyltransferase
AOS: Allene oxide synthase
APA: Alternative polyadenylation
AS: Alternative splicing
bHLH137: bHLH transcription factors
CCS: Circular consensus sequence
CCS1: Capsorubin synthase
CHS: Chalcone synthase
crtB: 15 cis-phytoene synthase
crtZ: Beta-carotene 3-hydroxylase
CYP1A1: Cytochrome P450 family 1 subfamily A polypeptide 1
CYP707A: (+)-abscisic acid 8′-hydroxylase
CYP75A: Flavonoid 3′,5′-hydroxylase
CYP75B1: Flavonoid 3′-monooxygenase
DEG: Differentially expressed gene
DFR: Bifunctional dihydroflavonol 4-reductase/flavanone 4-reductase
DWARF27: Beta-carotene isomerase
ETI: Effector-triggered immunity
F3H: Naringenin 3-dioxygenase
FLNC: Full-length non-chimeric
FMO: Dimethylaniline monooxygenase
FPKM: Fragments per kilobase of exon model per million reads mapped
GO: Gene ontology databases
GST: glutathione S-transferase
HSP90: Heat shock protein 90
IR: Intron retention
IRAK1: Interleukin-1 receptor-associated kinase 1
IRAK4: Interleukin-1 receptor-associated kinase 4
JA: Jasmonic acid
JAR1: Jasmonic acid resistance 1
KEGG: Kyoto Encyclopedia of Genes and Genomes pathway database
KOG: Eukaryotic orthologous groups databases
LAR: Leucoanthocyanidin reductase
LBP: Lipopolysaccharide-binding protein
LncRNA: Long non-coding rna
LOX: Lipoxygenase
MYB3R and MYB58: MYB transcription factors
MYC2: bHLH zip-type transcription factor
NCBI: National Center for Biotechnology Information
NCED: 9-cis-epoxycarotenoid dioxygenase
NGS: Next-generation sequencing
NPR1: Non-expressor of pathogenesis related gene 1
NR: NCBI non-redundant protein databases
OPCL1: OPC-8:0 CoA ligase 1
OPR: 12-oxophytodienoic acid reductase
PAL: Phenylalanine ammonia-lyase
PAMPs: Pathogen-associated molecular patterns
PR1: Pathogenesis related protein 1
PRR: Pattern recognition receptor
PRs: Pathogenesis-related proteins
PTI: PRR-triggered immunity
Pti6: ERF transcription factor
RBOH: Respiratory burst oxidase
SA: Salicylic acid
SAR: Systemic acquired resistance
SKIP: Exon skipping
SMRT: Single-molecule real-time
TF: Transcription factor
TGA4: bZIP transcription factor 4
ZEP: Zeaxanthin epoxidase
