# Supplementary material for: Candidate genes for grape white rot resistance based on SMRT and Illumina sequencing
Source: BMC Plant Biol. 2019 Nov 15;19:501. doi: 10.1186/s12870-019-2119-x (PMC6858721; doi:10.1186/s12870-019-2119-x)
Supplement: Supplementary file 1 — Additional file 1: Table S1. Illumina sequencing evaluation and mapping rate. [file 12870_2019_2119_MOESM1_ESM.docx]

| Sample | Total Reads | Total mapping rate | Length (bp) | Q30 (%) | GC (%) |
| --- | --- | --- | --- | --- | --- |
| VT1-1 | 51,909,446 | 43,281,445(83.4%) | 150 | 95.4; 90.7 | 46.7; 46.8 |
| VT1-2 | 56,565,014 | 46,174,280(81.6%) | 150 | 95.4; 90.4 | 46.7; 46.8 |
| VT1-3 | 61,691,728 | 50,336,439(81.6%) | 150 | 95.4; 91.0 | 46.8; 46.9 |
| VT2-1 | 53,324,776 | 42,175,442(79.1%) | 150 | 95.6; 91.4 | 46.7; 46.7 |
| VT2-2 | 62,777,654 | 50,640,553(80.7%) | 150 | 95.6; 91.5 | 46.5; 46.6 |
| VT2-3 | 43,567,460 | 35,080,909(80.5%) | 150 | 95.5; 91.9 | 46.4; 46.5 |
| ZX1-1 | 48,735,648 | 36,361,436(74.6%) | 150 | 95.6; 92.2 | 45.5; 45.6 |
| ZX1-2 | 74,760,242 | 56,112,974(75.1%) | 150 | 95.5; 91.3 | 46.5; 46.6 |
| ZX1-3 | 54,136,794 | 38,189,732(70.5%) | 150 | 95.5; 91.3 | 47.5; 47.6 |
| ZX2-1 | 42,440,120 | 32,249,106(76.0%) | 150 | 95.5; 90.0 | 46.6; 46.8 |
| ZX2-2 | 48,677,064 | 36,288,170(74.5%) | 150 | 95.4; 91.2 | 45.4; 45.5 |
| ZX2-3 | 44,789,388 | 33,233,827(74.2%) | 150 | 95.5; 89.5 | 46.8; 46.9 |

Table S1. Illumina sequencing evaluation and mapping rate.
