# Supplementary material for: Candidate genes for grape white rot resistance based on SMRT and Illumina sequencing
Source: BMC Plant Biol. 2019 Nov 15;19:501. doi: 10.1186/s12870-019-2119-x (PMC6858721; doi:10.1186/s12870-019-2119-x)
Supplement: Supplementary file 2 — Additional file 2: Table S2. Statistics analysis of SMRT data. [file 12870_2019_2119_MOESM2_ESM.docx]

Table S2. Statistics analysis of SMRT data.

| Library | Number |
| --- | --- |
| SMRT cells | 2 |
| Polymerase Reads | 393,168 |
| Subreads | 14,270,571 |
| Circular Consensus Sequence(CCS) | 64,7947 |
| Full-length Non-chimeric(FLNC) | 569,624 |
| Average Full-length Non-chimeric length | 1059 |
| FLNCs after stringent filtering | 493,335 |
