# Supplementary material for: Candidate genes for grape white rot resistance based on SMRT and Illumina sequencing
Source: BMC Plant Biol. 2019 Nov 15;19:501. doi: 10.1186/s12870-019-2119-x (PMC6858721; doi:10.1186/s12870-019-2119-x)
Supplement: Supplementary file 12 — Additional file 12: Table S3. Reads from Illumina sequencing alignment to the new constructed library [file 12870_2019_2119_MOESM12_ESM.docx]

| Sample | Total Reads | Total mapping rate |
| --- | --- | --- |
| VT1-1 | 51,909,446 | 43,724,992(84.23%) |
| VT1-2 | 56,565,014 | 46,410,236(82.05%) |
| VT1-3 | 61,691,728 | 51,135,844(82.89%) |
| VT2-1 | 53,324,776 | 42,933,426(80.51%) |
| VT2-2 | 62,777,654 | 51,804,920(82.52%) |
| VT2-3 | 43,567,460 | 35,575,950(81.66%) |
| ZX1-1 | 48,735,648 | 38,404,056(78.80%) |
| ZX1-2 | 74,760,242 | 59,656,496(79.80%) |
| ZX1-3 | 54,136,794 | 40,732,628(75.24%) |
| ZX2-1 | 42,440,120 | 34,742,098(81.86%) |
| ZX2-2 | 48,677,064 | 37,338,340(76.71%) |
| ZX2-3 | 44,789,388 | 36212638(80.85%) |

Table S3. Reads from Illumina sequencing alignment to the new constructed library.
