# Supplementary material for: Candidate genes for grape white rot resistance based on SMRT and Illumina sequencing
Source: BMC Plant Biol. 2019 Nov 15;19:501. doi: 10.1186/s12870-019-2119-x (PMC6858721; doi:10.1186/s12870-019-2119-x)
Supplement: Supplementary file 14 — Additional file 14: Table S4. Primers used for semi-quantitative PCR and qRT-PCR validation [file 12870_2019_2119_MOESM14_ESM.docx]

| Gene name | Forward primer | Reverse primer |
| --- | --- | --- |
| PR1 | CTACAACTCCAACTCATGCGTTG | GATCGTAGTTGCATGTGACGAAC |
| LOX | GGTAAATCACTCCGGTTAGAGCT | CTTTGACTCAACCCATGAACCAC |
| AOS | CGATCAGGCATCCTTCAACTTTC | CGCGTCGTAGAAGAAGTCATAGA |
| OPR | GGTACCTAGTGGACCAGTTCTTG | CAGCTCCAATCTCATCAGCAATG |
| OPCL1 | GCCTTGTTACTTACTCACCCAGA | GATCCTTCCTCAGAATCTTGCCT |
| ACOX | GATATATTCTGCCTGAGGGAGCG | GAGAAAGCTCTCCCCAAGTCTTC |
| JAR1 | GAGAGCATGAAATCATCGGTTGG | CTGGTTAGTGAGAATGGGAGAGG |
| PAL | CCGAGCATCAACTAAATCCATCG | GTTTCGGCTTCCAGAGAGATTTG |
| NPR1 | GTCTACCGAGGAAGGAAAACCTT | CAAATTCCTAGGCCTGATTGCAG |
| TGA4 | CGTTTGAGATGGAATATGGGCAC | TGTTCTCCACATGCCAGACATAA |
| Pti6 | TCTCACGCTATAAATACCCTCGC | AAGAGGAAGTCAGCAGGTATGTG |
| MYC2 | TCATTTACCAGAGGAGAAGGTGG | CAAGTTTCGACTCGATCACTGTG |
| MYB3R | GATCACTTCAACCCCCAAAAAGG | GAAAGTGTTCAGACCCTCCAGAT |
| MYB58 | GTCCTAACCCTAGCCCTAGTACT | GTCCAACATCTCCCAAAAACTGG |
| bHLH137 | GCTTATGCTGATACAACCACCAC | TTGGTAGGGCAGCTCAGTTTATT |
| β-actin | CAAGAGCGGAAACTGCAAAGA | AATGAGAGATGGCTGGAAGAGG |

Table S4. Primers used for semi-quantitative PCR and qPCR validation.
